# Exosomes derived from atorvastatin-modified bone marrow dendritic cells ameliorate experimental autoimmune myasthenia gravis by up-regulated levels of IDO/Treg and partly dependent on FasL/Fas pathway

**DOI:** 10.1186/s12974-016-0475-0

**Published:** 2016-01-12

**Authors:** Xiao-Li Li, Heng Li, Min Zhang, Hua Xu, Long-Tao Yue, Xin-Xin Zhang, Shan Wang, Cong-Cong Wang, Yan-Bin Li, Ying-Chun Dou, Rui-Sheng Duan

**Affiliations:** Department of Neurology, Shandong Provincial Qianfoshan Hospital, Shandong University, Jinan, 250014 People’s Republic of China; Department of Neurology, The Central Hospital of Taian, Taian, 271000 People’s Republic of China; Central Laboratory, Shandong Provincial Qianfoshan Hospital, Shandong University, Jinan, 250014 People’s Republic of China; School of Basic Medical Sciences, Jining Health School, Jining, 272000 People’s Republic of China; College of Basic Medical Sciences, Shandong University of Traditional Chinese Medicine, Jinan, 250355 People’s Republic of China

**Keywords:** Atorvastatin, Exosomes, Experimental autoimmune myasthenia gravis, IDO, FasL

## Abstract

**Background:**

Previously, we have demonstrated that spleen-derived dendritic cells (DCs) modified with atorvastatin suppressed immune responses of experimental autoimmune myasthenia gravis (EAMG). However, the effects of exosomes derived from atorvastatin-modified bone marrow DCs (BMDCs) (statin-Dex) on EAMG are still unknown.

**Methods:**

Immunophenotypical characterization of exosomes from atorvastatin- and dimethylsulfoxide (DMSO)-modified BMDCs was performed by electron microscopy, flow cytometry, and western blotting. In order to investigate whether statin-DCs-derived exosomes (Dex) could induce immune tolerance in EAMG, we administrated statin-Dex, control-Dex, or phosphate-buffered saline (PBS) into EAMG rats via tail vein injection. The tracking of injected Dex and the effect of statin-Dex injection on endogenous DCs were performed by immunofluorescence and flow cytometry, respectively. The number of Foxp3^+^ cells in thymuses was examined using immunocytochemistry. Treg cells, cytokine secretion, lymphocyte proliferation, cell viability and apoptosis, and the levels of autoantibody were also carried out to evaluate the effect of statin-Dex on EAMG rats. To further investigate the involvement of FasL/Fas in statin-Dex-induced apoptosis, the underlying mechanisms were studied by FasL neutralization assays.

**Results:**

Our data showed that the systemic injection of statin-Dex suppressed the clinical symptoms of EAMG rats. These statin-Dex had immune regulation functions in immune organs, such as the spleen, thymus, and popliteal and inguinal lymph nodes. Furthermore, statin-Dex exerted their immunomodulatory effects in vivo by decreasing the expression of CD80, CD86, and MHC class II on endogenous DCs. Importantly, the therapeutic effects of statin-Dex on EAMG rats were associated with up-regulated levels of indoleamine 2,3-dioxygenase (IDO)/Treg and partly dependent on FasL/Fas pathway, which finally resulted in decreased synthesis of anti-R97–116 IgG, IgG2a, and IgG2b antibodies.

**Conclusions:**

Our data suggest that atorvastatin-induced immature BMDCs are able to secrete tolerogenic Dex, which are involved in the suppression of immune responses in EAMG rats. Importantly, our study provides a novel cell-free approach for the treatment of autoimmune diseases.

## Background

Myasthenia gravis (MG) is an autoimmune disorder characterized by loss of acetylcholine receptor (AChR) on the postsynaptic membrane of neuromuscular junction, which results in defect in neuromuscular transmission and causes muscle weakness and fatigue [1]. The generation of autoantibodies to AChR is T cell dependent, in which CD4^+^ T cells help B cells to produce anti-AChR autoantibodies [2]. Experimental autoimmune myasthenia gravis (EAMG) can be induced in Lewis rats by immunization with *Torpedo* acetylcholine receptor (TAChR) or with a synthetic peptide corresponding to regions 97–116 of the rat AChR α subunit (R97–116 peptide). This EAMG model can mimic the human MG [3]. Currently used therapeutic drugs for MG include corticosteroids, immunosuppressants, antisense treatment (Monarsen, a synthetic antisense compound directed against the AChE gene) [4], and TNF-α receptor blocker (such as Etanercept) [5]. The mortality and morbidity of MG has decreased up to now [6]. Although the above mentioned drugs are effective in treating MG, their side effects are very severe. Thus, more effective drugs are still in urgent need.

Dendritic cells (DCs) are the professional antigen-presenting cells (APCs) in the immune system. Vaccine against DCs, a cellular treatment to induce immune tolerance, has been studied in different animal models. AChR-pulsed bone marrow DCs (BMDCs) could induce peripheral tolerance to EAMG through inhibiting the expression of B cell activating factor (BAFF) and the production of anti-AChR autoantibodies [7]. DCs modified with different cytokines in vitro or with RelB (an NF-κB family member that is responsible for DCs differentiation) specific small interfering RNA sequences have shown protective effects on the inhibition of the onset and progression of autoimmune diseases [8–11]. Statins, including atorvastatin, are 3-hydroxy-3-methyl-glutaryl coenzyme A (HMG-CoA) reductase inhibitors in the mevalonate pathway for cholesterol biosynthesis. Increasing evidences have shown that statins have immunomodulatory effects. The effects of statins on immune system include inhibiting the expression and secretion of pro-inflammatory cytokines [12], inhibiting T cell activation and proliferation [13], inhibiting the maturation and function of APCs [14]. Our previous study demonstrated that tolerogenic immature DCs could be induced by atorvastatin in vitro and these tolerogenic DCs successfully induced the immune tolerance in EAMG rats [15]. Thus, DCs vaccine may be an effective method for the treatment of autoimmune diseases. However, there are some limitations in DCs vaccine treatment. Among these limitations, the unstable characteristics of DCs vaccine in vitro is of most importance.

Exosomes are small particles (about 30–100 nm in size) secreted by different type of cells, such as DCs [16], T lymphocytes [17], and tumor cells [18]. In recent years, DCs-derived exosomes (Dex) have gained much attention in autoimmune diseases and tumors because they resemble the biology of cells from which they were derived [19]. There are many important regulatory molecules on Dex, such as MHC class I/II molecules, CD80, CD86, and CD40 (for antigen presentation and T cell stimulation) [20, 21]. Depending on the stage of maturation of DCs, there are at least two phenotypes of Dex, which are mature Dex and immature Dex. Mature Dex shows immunostimulatory effects [22] while immature Dex shows immunosuppressive effects [23]. It has been shown that exosomes derived from tumor peptide-pulsed DCs cause suppression of tumor growth in mice [24]. In a phase I study, Dex therapy results in immune activation and stability in advanced non-small cell lung cancer [25]. On the other hand, exosomes derived from immature BMDCs (iDex) ameliorated the progression of EAMG by reducing AChR-reactive lymphocyte proliferation, AChR antibody levels and pro-inflammatory cytokine levels [26]. IDex, which carries a moderate level of MHC class II and a low level of co-stimulatory molecules on their surface, prolong the intestinal allograft survival, suggesting that they may play important roles in immune regulation [27]. Indoleamine 2,3-dioxygenase (IDO) is an intracellular tryptophan-catabolizing rate-limiting enzyme that has immunosuppressive properties [28]. IDO can inhibit immune responses by enhancing apoptosis of T cells, inhibiting proliferation of T cells, and inducing the differentiation of naïve T cells into Treg cells [29–32]. It suggests that Dex over-carrying IDO could improve collagen-induced arthritis (CIA) and reduce inflammation in the delayed-type hypersensitivity (DTH) models [33]. Our previous study demonstrated that spleen-derived DCs modified with atorvastatin successfully induced the immune tolerance in EAMG rats. However, the effects of atorvastatin-modified DCs-derived exosomes (statin-Dex) on immune tolerance are still unknown.

In this study, we evaluated the effects of statin-Dex on EAMG and tried to investigate the mechanisms. We found that systemic administration of statin-Dex was able to suppress the clinical symptoms of EAMG rats. All the data suggest that atorvastatin-modified BMDCs can secrete tolerogenic Dex, and this may provide us a novel therapeutic approach for autoimmune diseases.

## Methods

### Animals and reagents

Six- to eight-week-old female Lewis rats (body weight 145–165 g) were purchased from Vital River Laboratories (Beijing, China) and kept under pathogen-free conditions at the local animal house. All rats were housed on a 12/12 light-dark schedule with water and food available ad libitum. All the experimental protocols were approved by the guidelines of the Animal Ethics Committee of Shandong University. Atorvastatin was kindly given by Beijing Garlin Pharmaceutical Co., Ltd (Beijing, China). R97–116 peptide (DGDFAIVKFTKVLLDYTGHI) was synthesized by AC Scientific, Inc. (Xian, China).

### Generation and culture of BMDCs

The tibia and femur bones were harvested from the ongoing EAMG rats (*n* = 40) on day 22 post immunization (p.i.) after removing the muscle tissue. Both ends of the bones were cut with a scissor, and the bone marrow was flushed into a new dish with phosphate-buffered saline (PBS) and passed through a cell strainer (70 μm; Becton Dickinson, Franklin Lakes, NJ, USA). Then, erythrocytes were osmotically lysed. Bone marrow cells were further enriched by differential adherence by incubating cells in 75 mm^2^ Falcon culture flasks (Becton Dickinson) in serum-free Roswell Park Memorial Institute (RPMI) 1640 (containing 2.05 mM glutamine, HyClone, Beijing, China) at 37 °C in a 5 % CO_2_ incubator. After 1 h 40 min, non-adherent cells were gently removed by swirling the flasks and aspirating the medium, then flasks were washed with serum-free medium to remove non-adherent cells. For Dex production, fetal calf serum (FCS; Gibco, Grand Land, NY, USA) depleted of contaminating vesicles and protein aggregates underwent overnight centrifugation at 110,000×*g*. Bone marrow cells were re-suspended in fresh complete medium containing 10 ng/ml recombinant rat granulocyte-macrophage colony-stimulating factor (rrGM-CSF) and 10 ng/ml recombinant rat IL-4 (rrIL-4) (Peprotech, Rocky Hill, NJ, USA). Same concentration of fresh medium was added on day 6. After 10 days of culture, the floating cells were collected as a DCs-enriched fraction. The DCs-enriched fraction showed approximately 80–85 % DCs by staining with anti-rat OX62 antibody (Becton Dickinson), which was considered as BMDCs. Then BMDCs were harvested for atorvastatin treatment.

For atorvastatin treatment, BMDCs were plated on six-well plates in complete culture medium without rrGM-CSF and rrIL-4. Atorvastatin dissolved in dimethylsulfoxide (DMSO, Sigma-Aldrich, St. Louis, MO, USA) was added to some wells (final concentration 10 μM, statin-BMDCs). The same volume of DMSO was added to the other wells (control-BMDCs). After incubation for 72 h in 37 °C, the supernatants were harvested for Dex purification. We found that approximately 3.5–8 μg of exosomes were obtained from the supernatants of 10^6^ BMDCs for 72 h culture.

### Dex purification

Dex were prepared from the cell culture supernatants of statin-BMDCs (statin-Dex) and control-BMDCs (control-Dex) by differential centrifugation as described by Raposo et al. [34]. Briefly, the culture supernatants were subjected to three successive centrifugations at 300×*g* (5 min), 1200×*g* (20 min), and 10,000×*g* (30 min) to eliminate cells and debris in the pellets, followed by centrifugation for 1 h at 100,000×*g*. To remove excess serum proteins, the Dex pellets were washed with a large volume of PBS, centrifuged at 100,000×*g* for 1 h, and re-suspended in PBS for further studies. Then, Dex were quantified by the K5600 MicroSpectroPhotoMeter (Beijing Kaiao Technology Development Co. Ltd, Beijing, China), and 1 μg was suspended in 20 μl of PBS for in vivo studies.

### Electron microscopy

Dex were purified by differential centrifugation, 10 μl loaded on a Formvar/carbon coated grid, negatively stained with 10 μl of neutral 1 % aqueous phosphotungstic acid, and viewed using JEOL-1210 computer-controlled, high-contrast, 120-kV transmission electron microscope.

### Flow cytometric analysis of Dex

For fluorescence-activated cell sorting (FACS) analysis, 30 μg of statin-Dex or control-Dex (or 30 μg FCS protein for negative control) were incubated with 10 μl of 4 μm-diameter aldehyde/sulfate latex beads (Invitrogen, Eugene, OR, USA) for 15 min at room temperature, followed by 2 h of gentle shaking in PBS. The reaction was stopped by incubation for 30 min in 100 mM glycine. Dex- or FCS-coated beads were washed three times in FACS wash buffer (3 % FCS and 0.1 % NaN_3_ in PBS) and re-suspended in 500 μl FACS wash buffer. Then, 10 μl of coated beads were incubated with phycoerythrin (PE)-conjugated anti-rat CD80 (BioLegend, San Diego, CA, USA), fluorescein isothiocyanate (FITC)-conjugated anti-rat CD86 (BioLegend), FITC-conjugated anti-rat MHC class II (eBioscience, San Diego, CA, USA), and FITC-conjugated anti-rat FasL antibodies (Santa Cruz Biotechnology, Santa Cruz, CA, USA), followed by washing and analysis using an FACScan (Beckman Coulter, Los Angeles, CA, USA).

### Western blotting

Statin-Dex and control-Dex samples (10 μg/lane) were loaded onto 12 % SDS-PAGE and subsequently transferred onto PVDF membrane (Millipore, Bedford, MA, USA). The membrane was blocked by incubation for 2 h at room temperature and incubated with mouse anti-rat IDO antibody (Santa Cruz Biotechnology) at 4 °C for overnight. After three washes with PBS containing 0.05 % (*v*/*v*) Tween20, the membrane was further incubated with goat anti-mouse horseradish peroxidase-labeled secondary antibody and visualized with the eECL detection system (Beijing CoWin Bioscience Co. Ltd, Beijing, China). Membranes were stripped and reblotted with an antibody for GAPDH as a loading control. Semiquantitative analysis of the protein density bands was quantified using the ImageJ software (National Institutes of Health, Bethesda, MD).

### Induction of EAMG and Dex administration

EAMG were induced by subcutaneous injection into both hind footpads with 200 μl inoculum containing 50 μg R97–116 peptide, 2 mg Mycobacterium tuberculosis (strain H37RA; Difco, Detroit, MI, USA) in incomplete Freund’s adjuvant (Sigma-Aldrich) on day 0 and were boosted with the same dose along the back on day 11 after the first immunization. The severity of the disease was scored by measuring muscular weakness in a blinded fashion from the beginning of the experiment every second day until day 43 p.i. Clinical scoring was based on the presence of tremor, hunched posture, muscle strength, and fatigability. Fatigability was assessed after exercise (repetitive paw grips on the cage grid) for 30 s. The severity of clinical symptoms was scored as follows [3, 15]: 0, normal strength and no abnormalities; 1, mildly decreased activity and weak grip or cry, more evident at the end of exercise; 2, clinical signs present before exercise (tremor, head down, hunched posture, weak grip); 3, severe clinical signs present before exercise, no grip, moribund; 4, dead. Rats with intermediate signs were assigned scores of 1.5, 2.5, or 3.5. Results are expressed as the mean of the evaluations recorded for each animal at each time point.

In our present study, the method and dosage of Dex administration were followed according to the studies by Kim et al. and Duchmann et al. [35, 36]. Statin-Dex and control-Dex were transferred into EAMG rats via tail vein injection at dose of 10 μg/rat on days 5 and 16 p.i. The control rats received the same volume of PBS.

### Tracking analysis and the effect of statin-Dex injection on endogenous DCs

To examine the mechanism of action of Dex in vivo, the tracking of injected Dex was examined in EAMG rats. Statin-Dex and control-Dex were labeled with PKH26 (red florescent cell linker; Sigma) as outlined by the manufacturer. After incubation with the linker, the labeled Dex were washed twice by ultracentrifugation and then intravenously injected into EAMG rats at dose of 10 μg/rat on day 5 p.i. Rats were killed on days 1 and 3 after injection, and the tissues (spleen, liver, thymus, popliteal, and inguinal lymph nodes) were obtained and frozen for histological analysis. Cryostat sections (6 μm) were made from these samples and examined by fluorescence microscopy (Olympus FSX100, Tokyo, Japan). Meanwhile, spleen-derived DCs from EAMG rats were prepared as previous methods [15]. Briefly, spleens were removed under aseptic conditions from EAMG rats on days 1 and 3 after injection of PKH26-labeled Dex, respectively. Mononuclear cell (MNC) suspensions from individual rats were obtained by grinding the spleens through cell strainers (Becton Dickenson) in medium. Then, erythrocytes were osmotically lysed. Spleen-derived DCs were further enriched by differential adherence by incubating cells in 25 mm^2^ Falcon culture flasks (Becton Dickinson) in serum-free RPMI 1640 (containing 2.05 mM glutamine, HyClone) at 37 °C in a 5 % CO_2_ incubator. After 1 h 40 min, non-adherent cells were gently removed by swirling the flasks and aspirating the medium, then flasks were washed with serum-free medium to remove non-adherent cells. New RPMI 1640 medium containing 1 % (*v*/*v*) minimum essential medium (MEM; Sigma-Aldrich), 50 μg/ml gentamicin (Shandong Lukang), and 10 % FCS (Gibco) (depleted of contaminating vesicles and protein aggregates underwent overnight centrifugation at 110,000×*g*) were added to the flasks. After 4-h incubation, floating cells were collected as a spleen-derived DCs-enriched fraction. Thereafter, we labeled these spleen-derived DCs (endogenous DCs) with FITC-conjugated anti-rat CD80 (AbD Serotec, Oxford, UK), FITC-conjugated anti-rat CD86 (BioLegend), and FITC-conjugated anti-rat MHC class II (eBioscience) for 30 min at 4 °C. Then, the percentages of PKH26-labeled endogenous DCs and the expression of CD80, CD86, and MHC class II on endogenous DCs were examined by flow cytometry (BD Biosciences, San Jose, CA, USA). The whole operation process was in dark.

To further investigate the mechanism of exogenous Dex injection on endogenous DCs, spleen-derived DCs from ongoing EAMG rats were cultured with PKH26-labeled statin-Dex and control-Dex in vitro, respectively. Following, these DCs were labeled with mouse anti-rat OX62 antibody and FITC-conjugated anti-mouse IgG antibody (green). Then, the labeled DCs were examined by fluorescence microscopy (Olympus FSX100).

### Immunohistochemistry

Segments of thymuses were dissected from EAMG rats. Paraffin tissue sections (5 μm) were deparaffinized and hydrated. The sections were treated with 0.3 % hydrogen peroxide for 20 min to block endogenous peroxidase activity. After three washes in PBS, the sections were boiled in citrate buffer for antigen retrieval and incubated overnight at 4 °C with mouse anti-rat Foxp3 antibody (1:100; eBioscience). Then, the sections were stained with horseradish peroxidase-conjugated goat anti-mouse secondary antibody (Zhongshan Goldenbridge Biotechnology, Beijing, China), followed by development with diaminobenzidine (DAB) substrate (Zhongshan Goldenbridge Biotechnology) to detect the number of Foxp3^+^ cells. As negative controls for immunostaining, the primary antibody was omitted. The tissue areas were measured by image analysis in five sections per thymus, and the results were expressed as the number of positive cells per square millimeter tissue section.

### Preparation of lymph node MNC

On day 43 p.i., EAMG rats were sacrificed and inguinal lymph nodes were removed under aseptic conditions. MNC suspensions were obtained by grinding the organs through cell strainers in serum-free medium. Then, cells were washed three times and re-suspended to 2 × 10^6^/ml in RPMI 1640 (HyClone) supplemented with 1 % (*v*/*v*) MEM (Sigma-Aldrich), 50 μg/ml gentamicin (Shandong Lukang Cisen), and 10 % (*v*/*v*) FCS (Gibco) for the following experiments.

### Flow cytometric analysis of lymph node MNC

Fixation and permeabilization of lymph node MNC were performed using the eBioscience Foxp3 Staining Buffer Set (eBioscience). FITC-conjugated anti-rat CD4, PE-conjugated anti-rat CD25, and PE-Cy5-conjugated anti-mouse/rat Foxp3 antibodies (all from eBioscience) were used for staining Treg cells according to the protocol recommended by eBioscience. For intracellular cytokines analysis, MNC were fixed with 2 % paraformadehyde for 20 min at 4 °C and permeabilized with 0.5 % saponin. Then, the cells were stained with PE-conjugated anti-rat IL-10 (Pharmingen, San Diego, CA, USA) and FITC-conjugated anti-rat TNF-α antibodies (BioLegend) for 30 min at 4 °C. For cell surface molecules, MNC suspensions in PBS containing 0.5 % BSA (Sigma-Aldrich) were incubated with FITC-conjugated anti-rat Fas antibody (Santa Cruz Biotechnology) for 30 min at 4 °C in the dark. For apoptosis analysis, MNC suspensions were washed twice with cold BioLegend cell staining buffer and then re-suspend cells in Annexin V Binding Buffer. Then, 100 μl of cell suspensions were transferred to another test tube. Five microliters of FITC-Annexin V and 10 μl of propidium iodide (PI) solution incubate were added for 15 min at room temperature in the dark. Then, the cells were analyzed by flow cytometry (BD Biosciences).

### Analysis of cell viability

Cell viability was assessed by Cell Counting Kit-8 (CCK-8). Briefly, MNC suspended in 200 μl aliquots containing 4 × 10^5^ cells were cultured in triplicates in flat-bottomed 96-well microtitre plates (Corning, NY, USA) in the presence of PBS, R97–116 peptide (10 μg/ml), or ConA (5 μg/ml). After 72 h of incubation, the cells were incubated with 10 μl CCK-8 for 4 h at 37 °C. Then, the absorbance was read at 450 nm on a microplate reader. Data are expressed as mean absorbance value (optical density (OD)) of samples ± standard deviation (SD).

### Determination of cytokines in culture supernatants by ELISA

Quantitative analysis of IFN-γ and IL-4 levels was performed by ELISA. MNC were cultured in the presence of R97–116 peptide (10 μg/ml). After 72 h incubation at 37 °C, the supernatants were collected and measured for IFN-γ and IL-4 by sandwich ELISA kits (both from eBioscience) following the manufacturer’s instructions. Determinations were performed in duplicate and the results were expressed as picogram per milliliter.

### ELISA for serum anti-R97–116 peptide IgG antibody and its subtype

A standard ELISA technique was used to detect R97–116 peptide specific antibody. Briefly, R97–116 peptide (5 μg/ml) was coated onto a flat-bottomed 96-well microtitre plates (Corning) in 0.1 M carbonate bicarbonate buffer (pH 9.6) overnight at 4 °C. Then, the plates were blocked with 200 μl of PBS containing 0.05 % Tween20 and 10 % FCS at 37 °C for 1.5 h. A total volume of 100 μl diluted serum samples (1:100 in PBS/0.05 % Tween20) were added and incubated for 2 h at room temperature. After washed, biotin rabbit anti-rat IgG (1:3000; Biosynthesis Biotechnology, Beijing, China), biotin anti-rat IgG1, IgG2a, and IgG2b (1:500; BioLegend) were added to the wells and incubated for 1 h. Then streptavidin-horseradish peroxidase (1:1000; Biosynthesis Biotechnology) was added, incubated at 37 °C for 30 min. Then, plates were washed with PBS containing 0.05 % Tween20 and followed by development with Tetramethylbenzidine (TMB) substrate (Tiangen Biotechnology, Beijing, China). Finally, plates were read at a wave length of 450 nm using a microplate ELISA reader. Each serum was tested in triplicate. Results are expressed as mean OD value of samples ± SD.

### FasL neutralization assays

To examine the effect of FasL on EAMG rats, statin-Dex were incubated with purified Hamster anti-Mouse and rat FasL blocking (0.1, 1, and 10 μg /ml) or purified Hamster IgG3, κ isotype control antibodies (10 μg/ml) (both from BD Pharmingen) as a negative control at 37 °C for 12 h followed by washing three times. Then, the neutralized statin-Dex were analyzed by flow cytometry, and the optimal neutralizing concentration was used for in vivo studies.

Following, statin-Dex blocked with anti-FasL or isotype antibodies were transferred into EAMG rats via tail vein injection at dose of 10 μg/rat on days 5 and 16 p.i., respectively. EAMG rats were scored from the beginning of the experiment every second day until day 43 p.i. The severity of clinical symptoms was scored as previous mentioned.

On day 43 p.i., EAMG rats were sacrificed and inguinal lymph nodes were removed under aseptic conditions. MNC suspensions were obtained and re-suspended to 2 × 10^6^/ml in RPMI 1640 (HyClone). For cell surface molecules, MNC suspensions were incubated with FITC-conjugated anti-rat Fas antibody (Santa Cruz Biotechnology) for 30 min at 4 °C. After staining, the cells were re-suspended in PBS and analyzed by flow cytometry (BD Biosciences). For apoptosis analysis, MNC suspensions were stained with FITC-Annexin V and PI solution. Then, the cells were analyzed by flow cytometry (BD Biosciences).

Meanwhile, serums in different time points (days 10, 21, 32, and 43 p.i.) were collected for detection of anti-R97–116 peptide IgG antibody and its subtype (IgG1, IgG2a, and IgG2b).

### Statistical analysis

The SPSS 17.0 computer program (SPSS Inc., Chicago, IL, USA) was used for all calculations and statistical evaluations. Differences were tested by two-tailed Student *t* test between two groups and by one-factor analysis of variance (ANOVA) followed by *post hoc* test among three groups. *p* < 0.05 was considered significant.

## Results

### Characterization of rat Dex

**Fig. 1:**
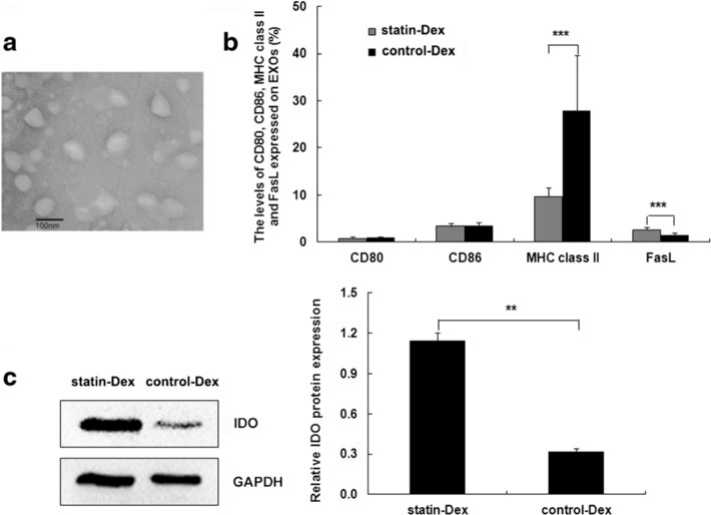
Characterization of rat Dex. To examine the composition of Dex, DCs were generated from the bone marrow precursors of ongoing EAMG rats (*n* = 40). Then, Dex were isolated from the culture medium of statin-BMDCs and control-BMDCs by differential centrifugation and characterized by electron microscopy (**a**), flow cytometry (**b**), and western blotting (**c**) (***p < 0.001)

To examine the composition of Dex, DCs were generated from the bone marrow precursors of ongoing EAMG rats and cultured in the presence of rrGM-CSF and rrIL-4. Then, Dex were isolated from the culture medium by differential centrifugation and characterized by electron microscopy, flow cytometry, and western blotting. Ultrastructural analysis of Dex by transmission electron microscopy showed a significant enrichment of the characteristic saucer-shaped vesicles, 30–100 nm in diameter (Fig. 1a). We further examined the surface proteins on the Dex by flow cytometry. Flow cytometric analysis showed that statin-Dex had lower level of MHC class II and higher level of FasL when compared with control-Dex (*p* < 0.001 for both comparisons) (Fig. 1b). Meanwhile, statin-Dex carried higher level of IDO protein when compared with control-Dex, confirmed by Western blotting analysis (Fig. 1c). Taken together, these data demonstrated that atorvastatin induced the production of tolerogenic Dex, and these tolerogenic Dex (statin-Dex) might play their immunoregulatory roles by lower level of MHC class II and high levels of FasL as well as IDO protein.

### Statin-Dex treatment suppress the development of EAMG

**Fig. 2:**
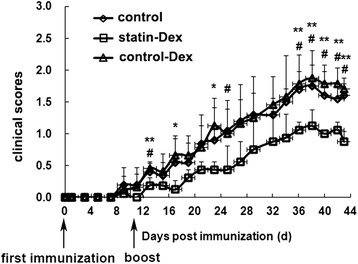
Statin-Dex treatment suppresses the development of ongoing EAMG in Lewis rats. Statin-Dex and control-Dex were transferred into EAMG rats via tail vein injection at dose of 10 μg/rat on days 5 and 16 p.i. The control rats received the same volume of PBS (*n* = 5 rats per group). The rats in statin-Dex group exhibited lower clinical scores when compared with rats in control-Dex group (*p < 0.05 and **p < 0.01) and control group (^#^ *p* < 0.05), while the clinical symptoms between control-Dex group and control group did not differ significantly. The data are expressed as mean ± SD and representative of two independent experiments

To investigate the immunoregulatory effects of statin-Dex, EAMG rats were injected intravenously with statin-Dex, control-Dex, or the same volume of PBS on days 5 and 16 p.i. The rats in statin-Dex group exhibited lower clinical scores when compared with the rats in control-Dex group (*p* < 0.05 and *p* < 0.01) and control group (*p* < 0.05). Moreover, the clinical symptoms between control-Dex group and control group did not differ significantly (Fig. 2). None of rats of the three groups died during the observation until day 43 p.i. These results showed that Dex with lower level of MHC class II and higher levels of FasL and IDO protein could contribute to the protective effects on EAMG.

### Dex tracking in vivo and in vitro

**Fig. 3:**
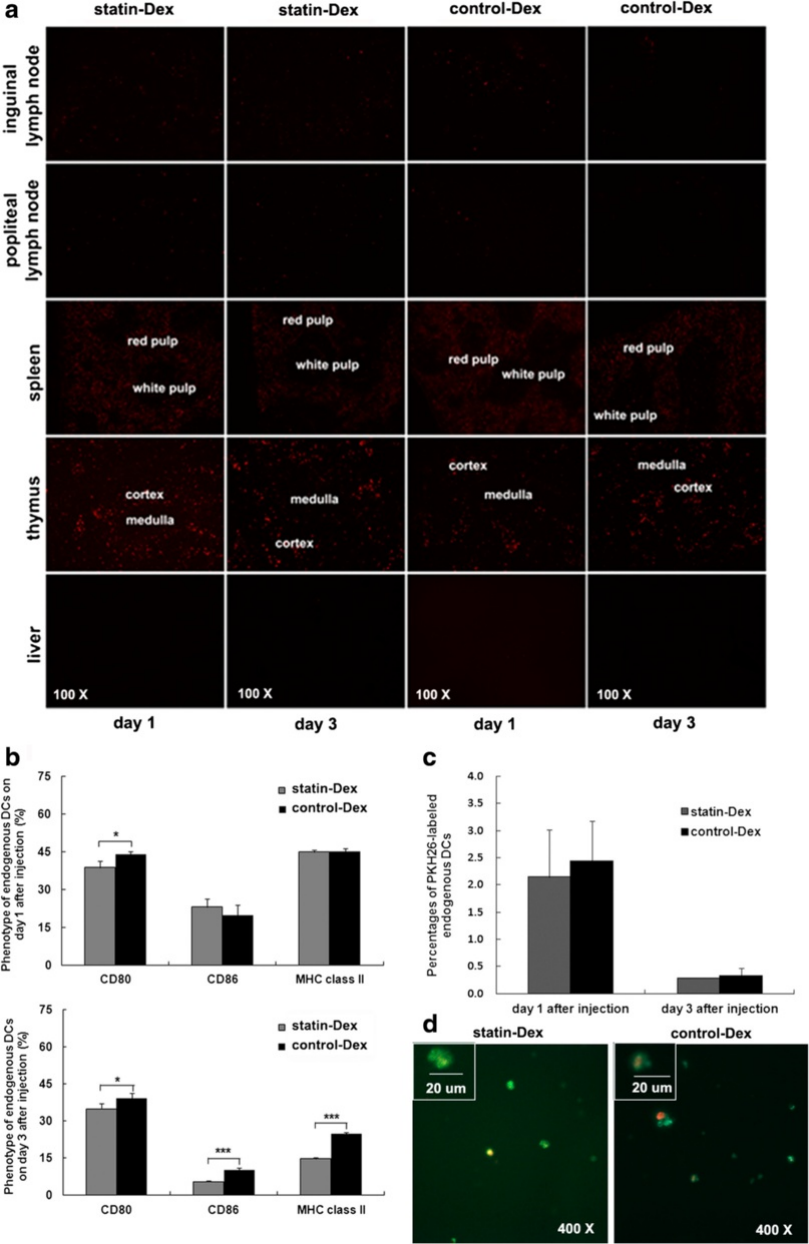
Distribution of Dex after injection into the EAMG rats and effects of statin-Dex injection on endogenous DCs. The PKH26-labeled (*red*) Dex were detected in the spleen, thymus, and popliteal and inguinal lymph nodes on days 1 and 3 after injection (**a**, original magnification ×100). The expression of CD80, CD86, and MHC class II on endogenous DCs on days 1 and 3 after injection were examined by FACS. The results showed that endogenous DCs from EAMG rats of statin-Dex group expressed lower level of CD80 on day 1 after injection and lower levels of CD80, CD86, and MHC class II on day 3 after injection when compared with those from control-Dex group. There were no significant differences for the levels of CD86 and MHC class II expressed on endogenous DCs from EAMG rats on day 1 after injection between statin-Dex group and control-Dex group (**b**). To further investigate the mechanism of exogenous Dex injection on endogenous DCs, spleen-derived DCs from ongoing EAMG rats were cultured with PKH26-labeled (*red*) statin-Dex and control-Dex in vitro, respectively. The percentages of PKH26-labeled endogenous DCs were examined by flow cytometry (**c**). Meanwhile, these DCs were labeled with mouse anti-rat OX62 antibody and FITC-conjugated anti-mouse IgG antibody (*green*). Then, the labeled DCs were examined by fluorescence microscopy. Both statin-Dex and control-Dex could be internalized by or fused with spleen-derived DCs in vitro (*red* stands for Dex, *green* stands for DCs, and *yellow* refers to overlap of red and green) (The diameter of spleen-derived DCs from rats is about 10–20 μm) (**d**, original magnification ×400). The results are expressed as mean ± SD (*n* = 5 rats per group) (*p < 0.05 and ***p < 0.001)

To determine the fate of Dex following injection, we labeled statin-Dex and control-Dex with PKH26 red fluorescent cell linker and intravenously injected them into EAMG rats on day 5 p.i. Then, we dissected the spleen, liver, thymus, popliteal, and inguinal lymph nodes on days 1 and 3 after injection. As shown in Fig. 3a, both labeled statin-Dex and control-Dex were detected in the spleen, thymus, and popliteal and inguinal lymph nodes, but were absent in liver. Moreover, less labeled Dex was found on day 3 after injection when compared with those on day 1 after injection. These labeled statin-Dex and control-Dex were mainly located in the red pulp of spleen and the cortex of thymus, while the labeled Dex could be found to be distributed diffusely within the lymph nodes. These characteristics of distribution indicated that statin-Dex could play their immunomodulatory effects in the immune organs of EAMG rats. In order to clarify the effects of exogenous Dex on endogenous DCs after injection in EAMG rats, we detected the expression of CD80, CD86, and MHC class II on endogenous DCs on days 1 and 3 after injection of PKH26-labeled exogenous Dex. Interestingly, the results showed that endogenous DCs from EAMG rats of statin-Dex group expressed lower level of CD80 on day 1 after injection and lower levels of CD80, CD86, and MHC class II on day 3 after injection when compared with those from control-Dex group (for CD80, *p* < 0.05 on days 1 and 3 after injection; for CD86 and MHC class II, *p* < 0.001 on day 3 after injection). There were no significant differences for the levels of CD86 and MHC class II expressed on endogenous DCs from EAMG rats on day 1 after injection between statin-Dex group and control-Dex group (Fig. 3b). Moreover, the percentages of PKH26-labeled endogenous DCs (both statin-Dex group and control-Dex group) on days 1 and 3 after injection were very low (both means <2.5 %), which indicated that only few exogenous Dex can be fused with and expressed on the surface of endogenous DCs (Fig. 3c). As shown in Fig. 3d, both statin-Dex and control-Dex could be internalized by or fused with spleen-derived DCs in vitro (*red* stands for Dex, *green* stands for DCs, and *yellow* refers to overlap of red and green).

### Statin-Dex treatment increase the number of Foxp3^+^ cells in thymus of EAMG rats

**Fig. 4:**
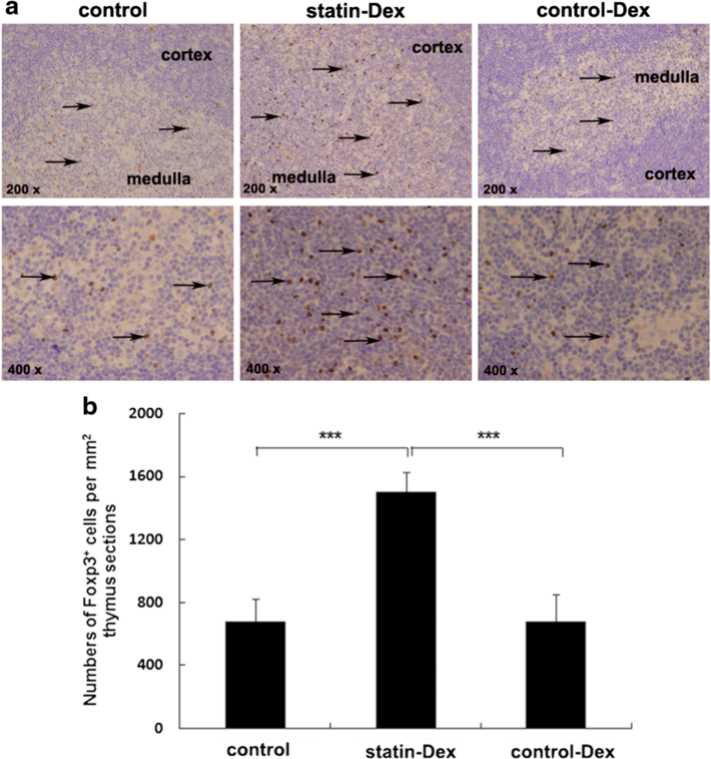
Statin-Dex treatment increases the number of Foxp3^+^ cells in thymus of EAMG rats. The number of Foxp3^+^ cells in thymus of EAMG rats was analyzed by immunohistochemistry. Statin-Dex, control-Dex, and PBS were transferred into EAMG rats via tail vein injection. Rats were sacrificed on day 43 p.i. **a** Representative micrographs show more Foxp3^+^ cells in the thymus in rats treated with statin-Dex compared to those in control-Dex and control rats, respectively. The *arrow* referred to typical Foxp3^+^ cells and the nucleus was filled with *brown*. Original magnification, ×200 and ×400. **b** The number of Foxp3^+^ cells per mm^2^ thymus sections is expressed as mean ± SD (*n* = 5 rats per group) (***p < 0.001)

Thymuses were taken from statin-Dex group, control-Dex group and control group rats on day 43 p.i. for immunohistochemistry analysis. We found that statin-Dex treatment increased the number of Foxp3^+^ cells in thymus when compared with control-Dex and PBS treatments (Fig. 4a). Further analysis showed that the number of Foxp3^+^ cells in statin-Dex group was higher than those in control-Dex group and control group (*p* < 0.001 for both comparisons), while there was no difference between control-Dex group and control group (Fig. 4b). These data indicated that the mild EAMG clinical scores in statin-Dex group were associated with the increased number of Foxp3^+^ cells in thymus.

### Statin-Dex treatment increase the number of CD4^+^Foxp3^+^ T cells in lymphocytes

**Fig. 5:**
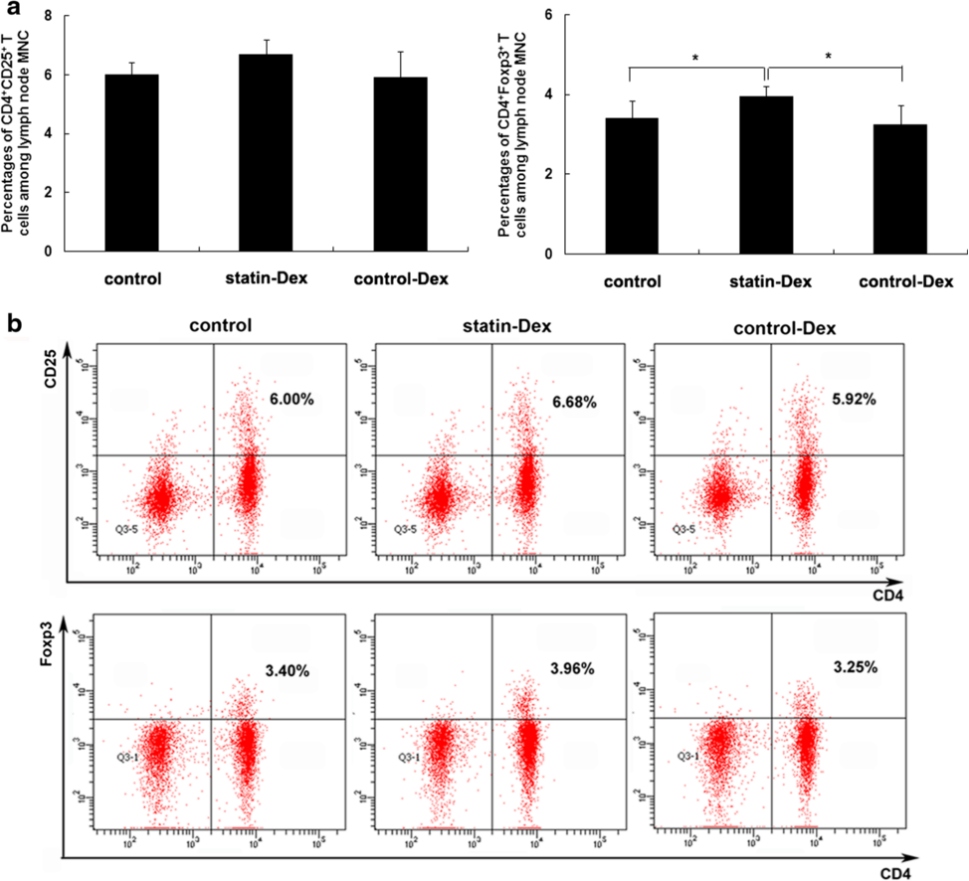
Statin-Dex treatment increase the number of CD4^+^Foxp3^+^ T cells in lymphocytes. Expressions of CD4^+^CD25^+^ T cells and CD4^+^Foxp3^+^ T cells among lymph node MNC in statin-Dex group, control-Dex group, and control group were detected by FACS. The results showed that statin-Dex treatment increased the percentage of CD4^+^Foxp3^+^ T cells among lymph node MNC when compared with control-Dex and PBS treatments, while there was no difference for the percentage of CD4^+^CD25^+^ T cells. Meanwhile, we did not observe difference in the percentages of CD4^+^CD25^+^ T cells and CD4^+^Foxp3^+^ T cells between control-Dex group and control group (**a**, **b**). The results are expressed as mean ± SD (*n* = 5 rats per group) (*p < 0.05)

To examine whether the effects of statin-Dex treatment were correlated with Treg cells in EAMG rats, MNC from lymph nodes on day 43 p.i. were stained with FITC-conjugated anti-rat CD4, PE-conjugated anti-rat CD25, and PE-Cy5-conjugated anti-mouse/rat Foxp3 antibodies. The results showed that statin-Dex treatment increased the percentage of CD4^+^Foxp3^+^ T cells among lymph node MNC when compared to control-Dex and PBS treatments (*p* < 0.05 for both comparisons), while there was no difference for the percentage of CD4^+^CD25^+^ T cells. Meanwhile, there was no difference for the percentages of CD4^+^CD25^+^ T cells and CD4^+^Foxp3^+^ T cells between control-Dex group and control group (Fig. 5a, b).

### Effects of statin-Dex treatment on the expression of intracellular cytokines in lymphocytes and on the lymphocyte proliferation

To gain insight into the mechanism underlying the effect of statin-Dex on EAMG, intracellular cytokines among lymph node MNC on day 43 p.i. were determined by flow cytometry. We found that there was no significant difference for the expression of IL-10^+^ and TNF-α^+^ cells among the three groups (data not shown). In order to investigate the antigen-specific lymphocyte response among statin-Dex group, control-Dex group, and control group, lymphocyte proliferation was measured after 72 h of culture in the presence of PBS, R97–116 peptide or ConA by using the CCK-8 assay. We observed that there was no difference among the three groups when MNC cultured with PBS and R97–116 antigen, although there was significant cell proliferation for ConA stimulation (data not shown). The data indicated that statin-Dex treatment on EAMG rats did not inhibit the proliferation of R97–116 antigen-specific T lymphocytes.

### Effects of statin-Dex treatment on the expression of Fas on lymph node MNC and analysis of apoptosis

**Fig. 6:**
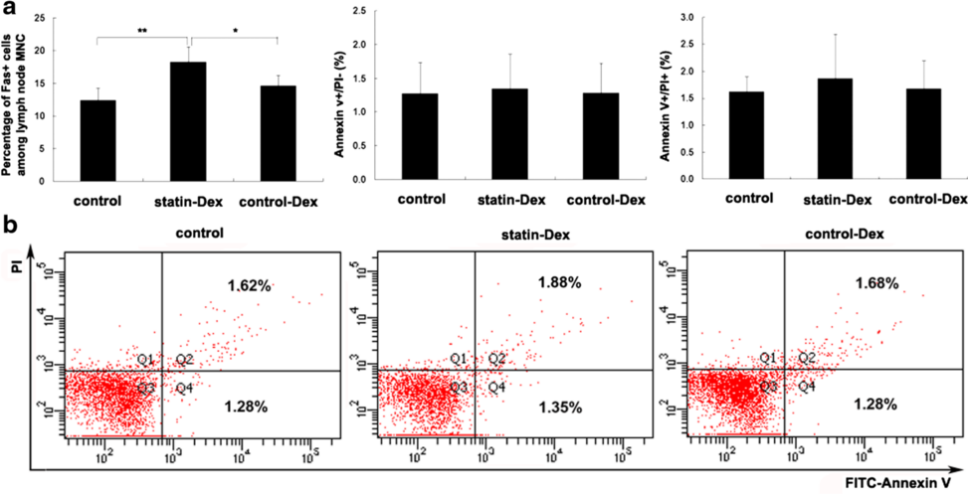
Effects of statin-Dex treatment on the expression of Fas on lymph node MNC and analysis of apoptosis. To examine whether statin-Dex could induce cell apoptosis through FasL/Fas pathway, the expression of Fas on lymph node MNC (**a**) and the distribution of apoptotic lymphocytes (**b**) were measured by flow cytometry. The results are expressed as mean ± SD (*n* = 5 rats per group) (*p < 0.05 and **p < 0.01)

In our study, we found that statin-Dex carried higher level of FasL; to examine whether statin-Dex could induce cell apoptosis through FasL/Fas pathway, the expression of Fas on lymph node MNC and the distribution of apoptotic lymphocytes were measured by flow cytometry. The results showed that statin-Dex treatment increased the percentage of Fas^+^ cells on lymph node MNC when compared with control-Dex and PBS treatments (*p* < 0.05 and *p* < 0.01, respectively), while there was no difference between control-Dex group and control group (Fig. 6a). For apoptosis analysis, on the image from the flow cytometry, cells in the upper-left portion (Q1), the upper-right portion (Q2), the lower-left portion (Q3), and the lower-right portion (Q4) represent dead cells, late-apoptotic cells + necrotic cells, viable cells, and early-apoptotic cells, respectively. In our present study, we observed that the numbers of apoptotic cells were not different among the three groups of rats, which indicated that FasL/Fas pathway might not be the main mechanism involved in the induction of cell apoptosis (Fig. 6b).

### Effects of statin-Dex treatment on the levels of IFN-γ and IL-4 in the culture supernatants

To further investigate the mechanisms of statin-Dex treatment on EAMG, we examined the levels of IFN-γ and IL-4 in culture supernatants of lymphocytes stimulated with R97–116 peptide. The results showed that statin-Dex treatment did not change the level of IFN-γ when compared with control-Dex and PBS treatments (data not shown). Moreover, there was almost no detectable IL-4 in culture supernatants of the three groups (data not shown). These data indicated that statin-Dex treatment did not disturb the balance of Th1 and Th2 responses in EAMG rats.

### Effects of statin-Dex treatment on the levels of anti-R97–116 peptide IgG antibody and its subtype in serum

**Fig. 7:**
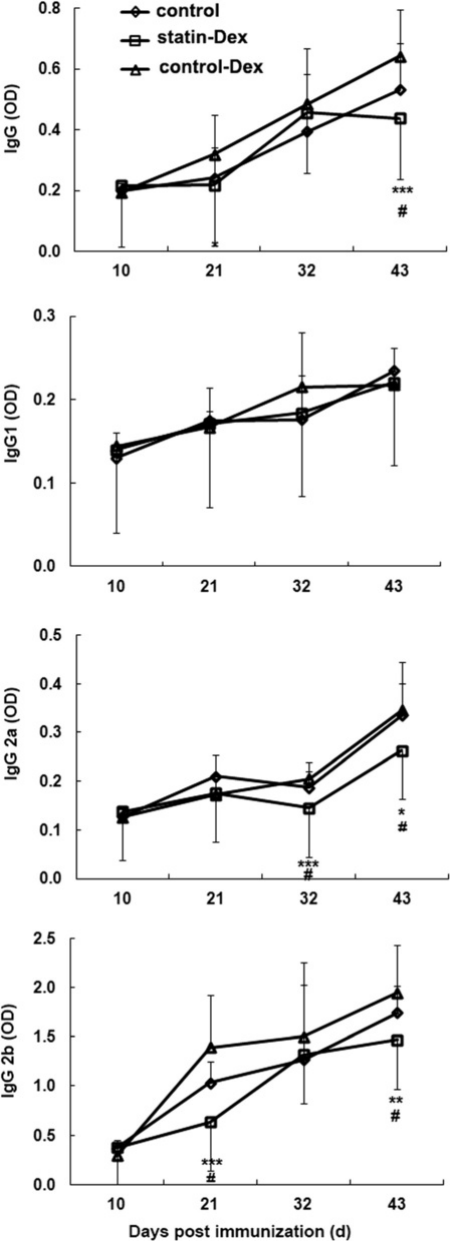
Effects of statin-Dex treatment on the levels of anti-R97–116 peptide IgG antibody and its subtype in serum. Rats treated with statin-Dex had lower levels of serum anti-R97–116 IgG, IgG2a, and IgG2b antibodies compared with control-Dex group rats (for IgG *p < 0.05 on day 21 p.i., ***p < 0.001 on day 43 p.i.; for IgG2a ***p < 0.001 on day 32 p.i., *p < 0.05 on day 43 p.i.; for IgG2b ***p < 0.001 on day 21 p.i., **p < 0.01 on day 43 p.i.) and control group rats (for IgG ^#^ *p* < 0.05 on day 43 p.i.; for IgG2a ^#^ *p* < 0.05 on days 32 and 43 p.i.; for IgG2b ^#^ *p* < 0.05 on days 21 and 43 p.i.). No significant difference in serum anti-R97–116 IgG antibody and its subtype was found between control-Dex group rats and control EAMG rats at different time points. Meanwhile, there was no statistical difference for anti-R97–116 IgG1 antibody among the three groups at different time points. Each serum was tested in triplicate. All results are expressed as mean ± SD (*n* = 5 rats per group)

In our present study, blood samples were collected on days 10, 21, 32, and 43 p.i. to determine the production of anti-R97–116 peptide IgG antibody and its subtypes IgG1, IgG2a, and IgG2b by ELISA. Rats treated with statin-Dex had lower levels of serum anti-R97–116 IgG, IgG2a, and IgG2b antibodies compared with control-Dex group rats (for IgG: *p* < 0.05 on day 21 p.i., *p* < 0.001 on day 43 p.i.; for IgG2a: *p* < 0.001 on day 32 p.i., *p* < 0.05 on day 43 p.i.; for IgG2b: *p* < 0.001 on day 21 p.i., *p* < 0.01 on day 43 p.i.) and control group rats (for IgG: *p* < 0.05 on day 43 p.i.; for IgG2a: *p* < 0.05 on days 32 and 43 p.i.; for IgG2b: *p* < 0.05 on days 21 and 43 p.i.). No significant difference in serum anti-R97–116 IgG antibody and its subtype was found between control-Dex group rats and control EAMG rats at different time points. Meanwhile, there was no statistical difference for anti-R97–116 IgG1 antibody among the three groups at different time points (Fig. 7).

### Benefits from statin-Dex treatment on EAMG rats are partly FasL/Fas pathway dependent

**Fig. 8:**
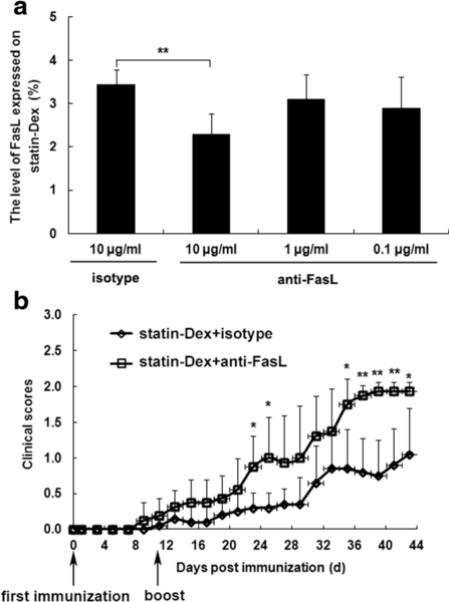
**a** The neutralized statin-Dex were analyzed by flow cytometry. Statin-Dex were incubated with purified Hamster anti-Mouse and rat FasL blocking (0.1, 1, and 10 μg/ml) or purified Hamster IgG3, κ isotype control antibodies (10 μg/ml). In the presence of anti-FasL blocking antibody (final concentration 10 μg/ml), the level of FasL expressed on statin-Dex were significantly decreased. The results are expressed as mean ± SD (*p < 0.05). **b** Effects of statin-Dex blocked with anti-FasL antibody on the development of EAMG in Lewis rats. Statin-Dex blocked with anti-FasL or isotype antibodies were transferred into EAMG rats via tail vein injection at dose of 10 μg/rat on days 5 and 16 p.i., respectively. The rats in statin-Dex with anti-FasL blocking antibody group exhibited higher clinical scores when compared with rats in statin-Dex with isotype control antibody group (*p < 0.05 and **p < 0.01). The data are expressed as mean ± SD (*n* = 6 rats per group)

**Fig. 9:**
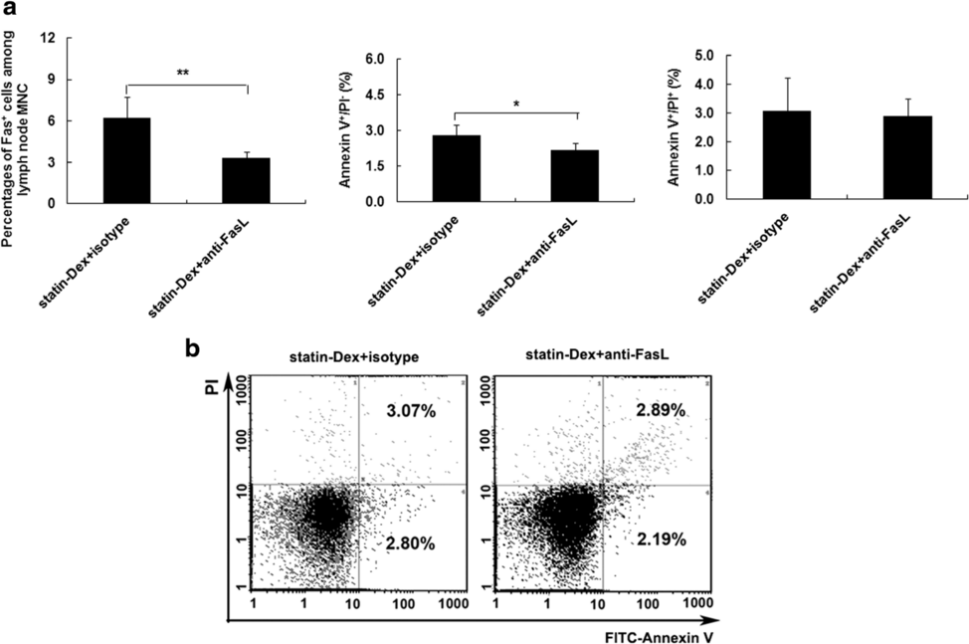
Effects of statin-Dex blocked with anti-FasL antibody on the expression of Fas on lymph node MNC and analysis of apoptosis. On day 43 p.i., EAMG rats were sacrificed and inguinal lymph nodes were removed under aseptic conditions. MNC suspensions were obtained. The percentage of Fas^+^ cells on lymph node MNC and the distribution of apoptotic lymphocytes were measured by flow cytometry (**a**, **b**). The results are expressed as mean ± SD (*n* = 6 rats per group) (*p < 0.05)

**Fig. 10:**
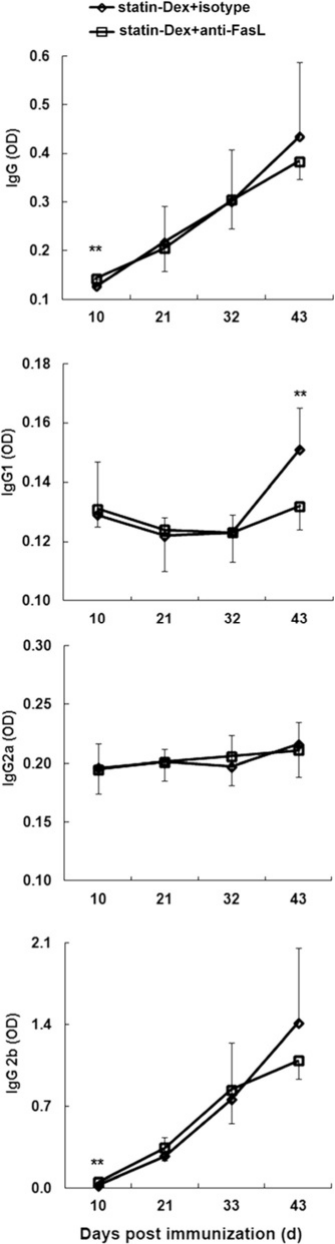
Effects of statin-Dex blocked with anti-FasL antibody on the levels of anti-R97–116 peptide IgG antibody and its subtype. Serums in different time points (days 10, 21, 32, and 43 p.i.) were collected for detection. Statin-Dex with anti-FasL blocking antibody treatment resulted in higher levels of serum anti-R97–116 IgG and IgG2b antibodies on day 10 p.i., and lower level of IgG1 antibody on day 43 p.i. when compared with statin-Dex with isotype control antibody treatment. Each serum sample was tested in triplicate. All results are expressed as mean ± SD (*n* = 6 rats per group) (**p < 0.01)

To further investigate the involvement of FasL in statin-Dex-induced apoptosis, we treated statin-Dex with anti-FasL blocking or isotype control antibody. In the presence of anti-FasL blocking antibody (final concentration: 10 μg/ml), the level of FasL on statin-Dex was decreased (Fig. 8a). Thereafter, statin-Dex blocked with anti-FasL or isotype antibody were transferred into EAMG rats via tail vein injection, respectively. The rats in statin-Dex with anti-FasL blocking antibody group exhibited higher clinical scores when compared with the rats in statin-Dex with isotype control antibody group (Fig. 8b). The results showed that there were significant decreases in both the level of Fas^+^ cells on lymph node MNC and the number of early-apoptotic cells in statin-Dex with anti-FasL blocking antibody group. Meanwhile, there was no significant difference for the number of late-apoptotic cells + necrotic cells between statin-Dex with anti-FasL blocking antibody group and statin-Dex with isotype control antibody group (Fig. 9a, b). These data suggested that anti-FasL blocking antibody, but not isotype control antibody, almost completely blocked statin-Dex treatment-induced cell apoptosis in EAMG rats. Meanwhile, statin-Dex with anti-FasL blocking antibody treatment resulted in higher levels of serum anti-R97–116 IgG, IgG2b antibodies on day 10 p.i. (*p* < 0.01 for both comparisons) and lower level of IgG1 antibody on day 43 p.i. (*p* < 0.01) when compared with statin-Dex with isotype control antibody treatment (Fig. 10). Taken together, our data indicated that statin-Dex treatment could induce cell apoptosis partly through FasL/Fas dependent mechanism, which would further lead to reduction of pathogenic anti-R97–116 IgG, IgG2a, and IgG2b antibodies.

## Discussion

In the present study, DCs were generated from bone marrow precursors of EAMG rats on day 22 p.i. Dex were prepared from the cell culture supernatants of statin-BMDCs and control-BMDCs, respectively. Compared with control-Dex, statin-Dex carried lower level of MHC class II and higher levels of FasL and IDO. We administrated statin-Dex, control-Dex, or PBS into EAMG rats via tail vein injection on days 5 and 16 p.i. Although the clinical symptoms were not significant on days 5 and 16 p.i., the immune response in vivo was activated in the early stage of disease. Therefore, we assumed that statin-Dex administrated in EAMG rats on days 5 and 16 p.i. played their therapeutic efficacy in ameliorating the disease. Our results confirmed that statin-Dex treatment suppressed clinical symptoms of EAMG compared with control-Dex and PBS treatments. After injection, these statin-Dex were detected in the spleen, thymus, and popliteal and inguinal lymph nodes. Furthermore, statin-Dex exerted their immunomodulatory effects in EAMG rats by decreasing the expression of CD80, CD86, and MHC class II on endogenous DCs. Finally, the therapeutic effects of statin-Dex on EAMG rats were associated with up-regulated level of IDO on statin-Dex and increased Treg cells in the thymus and lymph nodes. In addition, the effects of statin-Dex on EAMG were partly dependent on FasL/Fas pathway.

It is reported that Dex can transfer regulatory molecules to their corresponding acceptor cells. Exosomes containing MHC class II molecule as well as other proteins can attach to the surface of follicular DCs (FDCs). Thus, these FDCs passively acquire MHC class II molecule or other proteins from these donor exosomes [37]. Exosomes can be internalized by or fused with DCs and then processed by DCs for antigen presentation to CD4^+^ T cells [38]. Montecalvo et al. observed that exosomes first bound to the APCs surface and then internalized into endocytic vesicles of immature and mature DCs [39]. In our study, exogenous statin-Dex induced lower expression levels of CD80, CD86, and MHC class II on endogenous DCs when compared with control-Dex. Meanwhile, exogenous statin-Dex or control-Dex can be fused with endogenous DCs. We also found that both statin-Dex and control-Dex were captured and internalized by spleen-derived DCs in vitro. This indicates that the labeled Dex could be internalized by endogenous DCs and then processed for presentation of antigens to CD4^+^ T cells. Taken together, our data confirmed that exogenous statin-Dex played their roles by decreasing the expression of CD80, CD86, and MHC class II on endogenous DCs, which could further induce T cell anergy and immune tolerance in EAMG rats. These endogenous immature DCs may also increase the number of Treg cells in EAMG rats.

MHC class II molecule, which is expressed on APCs (such as DCs and B cells), is required for presenting antigen peptides to T cells. DCs- or B cell-derived exosomes carrying MHC class II peptide complexes could transfer allogeneic antigen to T cells or other APCs during the induction of immune responses and tumor rejection [40, 41]. Exosomes from class II trans-activator (CIITA)-transduced murine melanoma cells contain a large amount of MHC class II and exhibit greater effects on tumor regression [42]. Circulating MHC class II^+^ exosomes in tumor-bearing hosts are able to suppress the immune responses specific to tumor antigens [43]. As compared with mDex, iDex shows less enriched in MHC class II and CD86 as well as ICAM-1 and less potent for antigen-specific T cell activation [44]. In our study, statin-Dex showed lower levels of MHC class II when compared with control-Dex. However, our data did not provide evidence for the inhibitory effects of statin-Dex treatment on proliferation of R97–116 antigen-specific T lymphocytes. This suggests that MHC class II molecule derived from statin-Dex might not mainly be responsible for presentation of antigens to T cells in EAMG. Meanwhile, statin-Dex treatment did not change the percentage of IL-10^+^ and TNF-α^+^ cells and the level of IFN-γ compared with control-Dex and PBS treatments. There was almost no detectable IL-4 in culture supernatants in all the three groups. This indicates that statin-Dex treatment may not disturb the balance between Th1 and Th2 responses in EAMG rats. Consistently, Mallegol et al. have shown that DCs-derived MHC class II molecule, but not exosome-derived MHC class II molecule, is responsible for presentation of antigens to T cells [45]. Therefore, the mechanisms underlying the effects of statin-Dex on EAMG still need further investigation.

Recently, the immunosuppressive roles of IDO have been widely investigated in rheumatoid arthritis (RA) [46], tumor [47], transplantation [48], and EAE [49]. Several studies have demonstrated the association between the expression of IDO in DCs and the induction of Treg cells in different physiological and pathological settings. It is reported that the induction of IDO activity in DCs (IDO-competent DCs) promoted the differentiation of naïve CD4^+^ T cells into Foxp3^+^ Treg cells and induced the activation and regulatory function of quiescent Treg cells [50, 51]. Gut CD103^+^ DCs with IDO expression in the gut is reported to induce Foxp3^+^ Treg cells differentiation and inhibit IL-17 production, thus contributing to intestinal homeostasis [52]. Enhanced expression of IDO in splenic IDO^+^ CD11b^+^ DCs is associated with impaired proliferation of type II collagen (CII)-reactive T cells, and with CII-activated increase in converted Treg cells and Treg/Th17 ratio [53]. However, it is still unknown whether exosomes over-carrying IDO could activate Treg cells. In our present study, statin-Dex exhibited higher level of IDO when compared with control-Dex, suggesting that statin-Dex over-carrying IDO may increase the number and/or function of Treg cells and further be involved in immunomodulation. Consistent with our speculation, EAMG rats administrated with statin-Dex showed significantly increased numbers of Foxp3^+^ cells in the thymus and CD4^+^Foxp3^+^ T cells in MNC from the lymph nodes when compared with control-Dex group and control group. Therefore, we conclude that these Treg cells play important roles in maintaining immunologic tolerance of EAMG.

Ligation of FasL to Fas triggers the activation of caspases (such as caspase-8, caspase-3, caspase-6, and caspase-7) and eventually leads to apoptosis, even to cell death [54]. It has been demonstrated that FasL expressed on exosomes released by tumor cells could induce CD8^+^ T cell apoptosis [55]. The suppressive ability of the DC/FasL-derived exosomes on the DTH response is dependent not only on FasL in the DCs-derived exosomes but also on the presence of Fas in the host mice [36]. At the beginning of the neoplastic process, tumor cell-derived exosomes selectively induce apoptosis of antigen-specific T cells, which in turn up-regulate expression of Fas on the T cells. Thus, the FasL expressed on exosomes could induce apoptosis of T cells by binding with Fas [56]. These results indicate that the interaction between FasL and Fas is dependent on each other’s existence. In our study, statin-Dex carried higher level of FasL compared with control-Dex and statin-Dex treatment increased the number of Fas^+^ cells on lymph node MNC. However, our data showed that there was no significant difference among the three groups in the numbers of apoptotic cells. Consistent with our results, Kim et al. found that exosomes from DC/FasL did not increase the level of apoptosis in the lymph nodes and spleen, and the suppression of the DTH response required Fas in the recipient mice [23]. Reportedly, death of peripheral T cells is mainly mediated by two factors: the death receptor Fas (which is part of the extrinsic pathway) and the pro-apoptotic molecule BIM (BCL-2-interacting mediator of cell death) (which is part of the intrinsic pathway). Recent studies have proposed that BIM plays a major role in the induction of T cell apoptosis. It has been shown that BIM is crucial for the termination of the immune response against acute infection with herpes simplex virus (HSV) [57]. In contrast, loss of Fas does not affect the immune response to acute HSV infection [57]. Our results showed that although Fas/FasL was increased, statin-Dex treatment did not increase apoptosis of T cells in EAMG rats. This indicates that Fas/FasL extrinsic pathway might not play a major role in the apoptosis of T cells. It has been reported that administration of exosomes derived from DC/IL-4 was able to modulate the activity of APCs and T cells in vivo through a MHC class II and partly FasL/Fas-dependent mechanism, resulting in effective treatment of CIA and suppression of the DTH response [36]. In our study, statin-Dex with anti-FasL blocking antibody group had decreased level of Fas^+^ cells in lymph node MNC and decreased number of early-apoptotic cells. Meanwhile, statin-Dex with anti-FasL blocking antibody treatment induced higher levels of serum anti-R97–116 IgG and IgG2b antibodies and lower level of IgG1 antibody. And, the rats in statin-Dex with anti-FasL blocking antibody treatment group exhibited more severe clinical symptoms when compared with the rats in statin-Dex with isotype control antibody treatment group. Collectively, these results suggest that the immune tolerance induced by statin-Dex treatment on EAMG is partly dependent on the FasL/Fas pathway.

In both MG and EAMG, antibodies to AChR are directly responsible for the destruction of the muscle endplate. Meanwhile, CD4^+^ T cells also can help B cells to produce anti-AChR antibodies. Results in the present study confirmed that statin-Dex treatment decreased the levels of serum anti-R97–116 IgG, IgG2a, and IgG2b antibodies compared with control-Dex and PBS treatments. The changes in the levels of serum antibodies were consisted with the clinical symptoms of EAMG. The tolerogenic Dex may induce hyporesponsiveness of T cells through DCs. However, the exact mechanism underlying this effect of Dex is still unclear. In our present study, statin-Dex migrated to the immune organs after injection into the EAMG rats and was captured by endogenous DCs. These statin-Dex may play roles in the induction of immune tolerance in EAMG rats by decreasing expression of CD80, CD86, and MHC class II on endogenous DCs. The endogenous immature DCs, which lacks co-stimulatory molecules of CD80, CD86, and MHC class II, could induce T cells entering into a state of anergy or apoptosis and further result in decrease of the synthesis of anti-R97–116 IgG and its subtype antibodies. In addition, the decreased anti-R97–116 IgG and its subtype antibodies also can be due to up-regulated Treg cells.

Accumulating reports have shown that statins can cause muscle pain and weakness, which may induce or exacerbate MG [58, 59]. The underlying mechanisms are associated with decreased mitochondrial activity mediated by impaired coenzyme Q10 [60], as well as elevated AChR antibody levels after statin treatment [61]. Our study used statin-Dex rather than statin itself to treat EAMG rats, which circumvented the statin-induced muscular adverse effects in MG.

## Conclusions

Our study demonstrated that statin-Dex had immune regulation functions in immune organs. Meanwhile, these tolerogenic Dex played roles on the induction of immune tolerance in EAMG rats by inhibiting the expression of CD80, CD86, and MHC class II on endogenous DCs. Importantly, their inhibitory effects were associated with up-regulated level of IDO/Treg and were partly dependent on FasL/Fas pathway, which finally resulted in decreased synthesis of anti-R97–116 IgG, IgG2a, and IgG2b antibodies. However, the immune suppression of statin-Dex on EAMG was not antigen-specific. In conclusion, our data showed that statin-Dex modulated immune responses of EAMG and that statin-Dex might be useful for the treatment of human autoimmune diseases.
