# Correction to: Exosomes derived from atorvastatin-modified bone marrow dendritic cells ameliorate experimental autoimmune myasthenia gravis by up-regulated levels of IDO/Treg and partly dependent on FasL/Fas pathway

**DOI:** 10.1186/s12974-019-1503-7

**Published:** 2019-06-06

**Authors:** Xiao-Li Li, Heng Li, Min Zhang, Hua Xu, Long-Tao Yue, Xin-Xin Zhang, Shan Wang, Cong-Cong Wang, Yan-Bin Li, Ying-Chun Dou, Rui-Sheng Duan

**Affiliations:** 1 Department of Neurology, Shandong Provincial Qianfoshan Hospital, Shandong University, Jinan, 250014 People’s Republic of China; 2 Department of Neurology, The Central Hospital of Taian, Taian, 271000 People’s Republic of China; 3 Central Laboratory, Shandong Provincial Qianfoshan Hospital, Shandong University, Jinan, 250014 People’s Republic of China; 4 School of Basic Medical Sciences, Jining Health School, Jining, 272000 People’s Republic of China; 5 College of Basic Medical Sciences, Shandong University of Traditional Chinese Medicine, Jinan, 250355 People’s Republic of China

**Correction to: J Neuroinflammation**

**https://doi.org/10.1186/s12974-016-0475-0**

After the publication of the original article [1], it came to the authors’ attention that there was an error in the originally published version of Fig. 5b. The image of CD4^+^CD25^+^ T cells of the statin-Dex group was unintentionally replaced with the image of CD4^+^CD25^+^ T cells from the control group. The correct version of Fig. 5b is published in this Erratum.

**Fig. 5:**
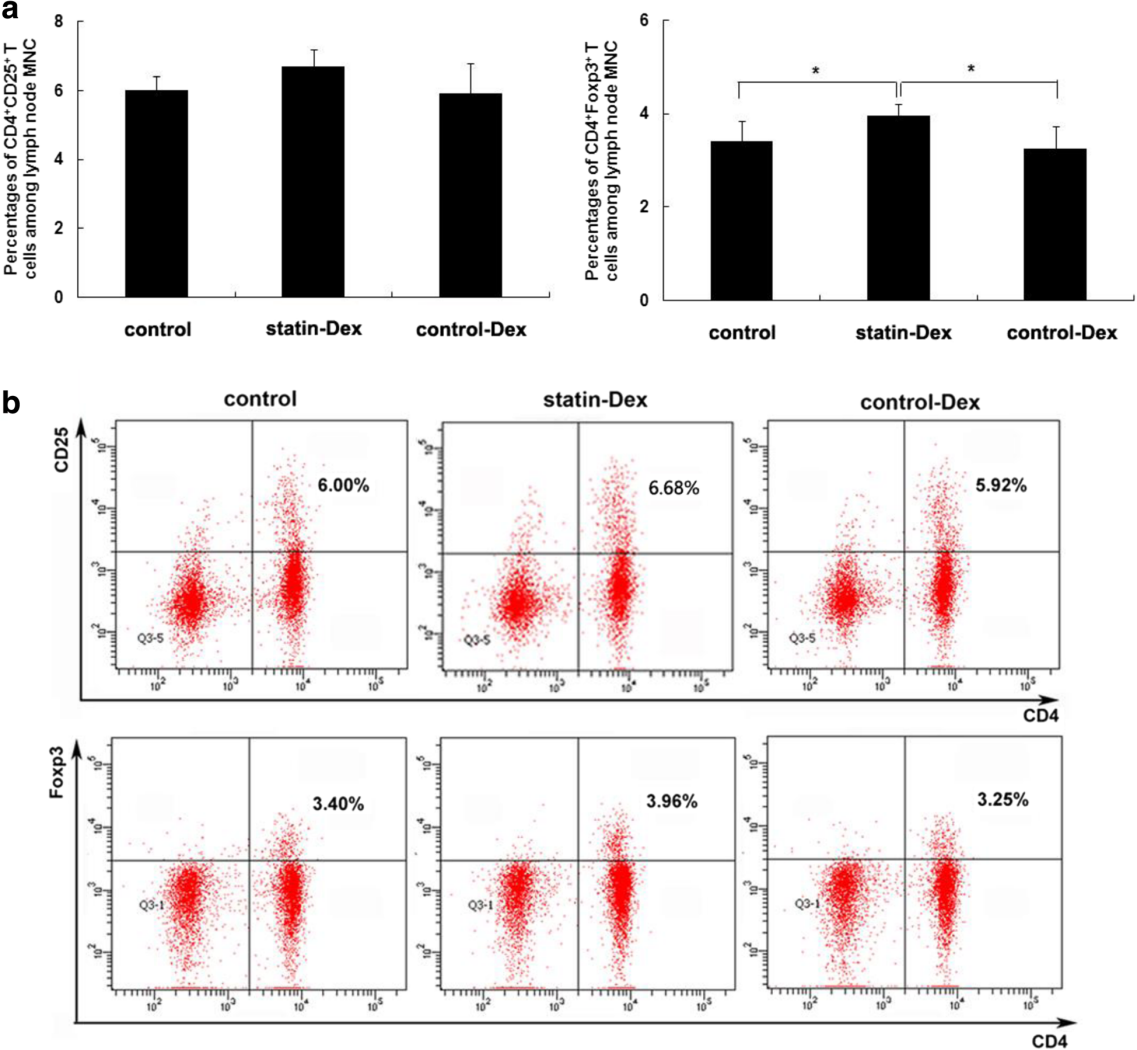
Statin-Dex treatment increase the number of CD4^+^Foxp3^+^ T cells in lymphocytes. Expressions of CD4^+^CD25^+^ T cells and CD4^+^Foxp3^+^ T cells among lymph node MNC in statin-Dex group, control-Dex group, and control group were detected by FACS. The results showed that statin-Dex treatment increased the percentage of CD4^+^Foxp3^+^ T cells among lymph node MNC when compared with control-Dex and PBS treatments, while there was no difference for the percentage of CD4^+^CD25^+^ T cells. Meanwhile, we did not observe difference in the percentages of CD4^+^CD25^+^ T cells and CD4^+^Foxp3^+^ T cells between control-Dex group and control group (**a**, **b**). The results are expressed as mean ± SD (*n* = 5 rats per group) (*p < 0.05)

## References

[1] Li X-L, Li H, Zhang M, Xu H, Yue L-T, Zhang X-X, Wang S, Wang C-C, Li Y-B, Dou Y-C, Duan R-S (2016). Exosomes derived from atorvastatin-modified bone marrow dendritic cells ameliorate experimental autoimmune myasthenia gravis by up-regulated levels of IDO/Treg and partly dependent on FasL/Fas pathway. J Neuroinflammation.

